# Safety and Efficacy of Kartigen^®^ in Treating Cartilage Defects: A Randomized, Controlled, Phase I Trial

**DOI:** 10.3390/polym13183029

**Published:** 2021-09-07

**Authors:** Yen-Liang Liu, Chun-Che Yen, Tzu-Shang Thomas Liu, Chih-Hung Chang, Tiffany Ting-Fang Shih, Jyh-Horng Wang, Ming-Chia Yang, Feng-Huei Lin, Hwa-Chang Liu

**Affiliations:** 1 Master Program for Biomedical Engineering, College of Biomedical Engineering, China Medical University, Taichung 406040, Taiwan; allen.liu@cmu.edu.tw; 2 Graduate Institute of Biomedical Sciences, College of Medicine, China Medical University, Taichung 406040, Taiwan; 3 Kartigen Biomedical Inc., Taipei 100047, Taiwan; cyenslk@gmail.com; 4 Southern California Bone and Joint Clinic, Apple Valley, CA 92307, USA; redcometsports@gmail.com; 5 Department of Orthopaedic Surgery, Far Eastern Memorial Hospital, New Taipei 220216, Taiwan; orthocch@mail.femh.org.tw; 6 Graduate School of Biotechnology and Bioengineering, Yuan Ze University, Taoyuan 320315, Taiwan; 7 Department of Medical Imaging and Radiology, National Taiwan University Hospital, Taipei 100225, Taiwan; ttfshih@ntu.edu.tw; 8 Department of Orthopaedic Surgery, National Taiwan University Hospital, Taipei 100225, Taiwan; jhwang@ntuh.gov.tw; 9 Biomedical Technology and Device Research Laboratories, Industrial Technology Research Institute, Hsinchu 310401, Taiwan; s1979329@gmail.com; 10 Department of Biomedical Engineering, College of Engineering, National Taiwan University, Taipei 106319, Taiwan; double@ntu.edu.tw; 11 Department of Orthopaedic Surgery, Taiwan Adventist Hospital, Taipei 105404, Taiwan

**Keywords:** cartilage defect, knee, Kartigen^®^, chondrocyte precursors, stem cell therapy

## Abstract

Here, we aimed to investigate the safety and preliminary efficacy of Kartigen^®^, a matrix with autologous bone marrow mesenchymal stem cell-derived chondrocyte precursors embedded in atelocollagen. As a surgical graft, Kartigen^®^ was implanted onto the cartilage defects at the weight-bearing site of the medial femoral condyle of the knee. Fifteen patients were enrolled and stratified into two groups, undergoing either Kartigen^®^ implantation (*n* = 10) or microfracture (control group, *n* = 5). The primary endpoint was to evaluate the safety of Kartigen^®^ by monitoring the occurrence of adverse events through physician queries, physical examinations, laboratory tests, and radiological analyses for 2 years. There were no infections, inflammations, adhesions, loose body, or tumor formations in the Kartigen^®^-implanted knees. The preliminary efficacy was assessed using the International Knee Documentation Committee (IKDC) score, visual analog scale, and second-look arthroscopy. The postoperative IKDC scores of the Kartigen^®^ group significantly improved in the 16th week (IKDC = 62.1 ± 12.8, *p* = 0.025), kept increasing in the first year (IKDC = 78.2 ± 15.4, *p* < 0.005), and remained satisfactory in the second year (IKDC = 73.6 ± 13.8, *p* < 0.005), compared to the preoperative condition (IKDC = 47.1 ± 17.0), while the postoperative IKDC scores of the control group also achieved significant improvement in the 28th week (IKDC = 68.5 ± 6.1, *p* = 0.032) versus preoperative state (IKDC = 54.0 ± 9.1). However, the IKDC scores decreased in the first year (IKDC = 63.5 ± 11.6) as well as in the second year (IKDC = 52.6 ± 16.4). Thirteen patients underwent second-look arthroscopy and biopsy one year after the operation. The Kartigen^®^ group exhibited integration between Kartigen^®^ and host tissue with a smooth appearance at the recipient site, whereas the microfracture group showed fibrillated surfaces. The histological and immunohistochemical analyses of biopsy specimens demonstrated the columnar structure of articular cartilage and existence of collagen type II and glycosaminoglycan mimic hyaline cartilage. This study indicates that Kartigen^®^ is safe and effective in treating cartilage defects.

## 1. Introduction

Cartilage defects are highly prevalent joint disorders and leading causes of disability and chronic pain in the world [1]. Articular cartilage defects are mainly attributed to trauma, osteoarthritis, osteonecrosis, and osteochondritis dissecans. Chondral lesions were found in around 60% of the patients undergoing knee arthroscopy [2,3,4].

Back in 1743, Hunter described the challenge of cartilage repair, stating that “once the cartilage is destroyed, it never recovers [5,6].” His observation still holds today. The avascular characteristics of cartilage constrain its self-regeneration from injury. If left untreated, the damaged cartilage gradually progresses into severe osteoarthritis. Through decades of effort, multiple surgical treatments have been developed to promote cartilage healing, such as abrasion arthroplasty [7], microfracture [8,9], and mosaicplasty [10,11]. However, these surgical approaches are usually associated with fibrocartilage formation [12,13], limited tissue sources [14], and donor-site morbidity [15], and its long-term efficacy remains controversial [16].

Cell therapy for cartilage repair was proposed in the 1980s by Robert Langer and Charles Vacanti using the approaches of tissue engineering. They clearly defined that a fine reconstruction of cartilage defect must include selected cells for transplantation, excipients, which are seeded with selected cells, and functional restoration of defect areas as before [17]. The cellular therapeutic innovation was realized in 1994. Autologous chondrocyte implantation (ACI) was introduced to treat cartilage defects in the knee [18]. In the past two decades, ACI has demonstrated its efficacy for knee osteoarthritis. Two systematic reviews concluded that, relative to microfracture and mosaicplasty, ACI may be the best option for large defects in active young patients who have had the symptoms for a short period and have not undergone a chondral surgery before [13,19]. In addition, a scaffold-based ACI, matrix-induced autologous chondrocyte implantation (MACI), outperformed microfracture in a 2-year randomized study [20]. Still, only a few products are available on the market: Carticel (FDA-approved in 1997), Chondron (South Korea MFDS-approved in 2001), and MACI (FDA-approved in 2016). The scarcity implies that some limitations remain, such as the limited source of chondrocytes, donor site morbidity, uncertain hyaline cartilage formation, low recovery of the recipient site, and questionable longevity of these implants or their derivative tissues [21].

The discovery of adult stem cells aroused a paradigm shift in regenerative medicine. The features of self-renewal and multipotency of stem cells make them ideal cell sources for cellular therapy. A variety of stem cell-based therapeutic innovations have been developed using mesenchymal stem cells (MSCs) derived from bone marrow [22], adipose tissue [23], synovium [24], peripheral blood [25], or periosteum [26]. Several clinical trials have demonstrated the safety and therapeutic efficacy of autologous MSC implantation for cartilage repair [27,28,29,30]. However, the implantation of undifferentiated MSCs cannot guarantee certain chondrogenesis in vivo, which might lead to heterogeneity of regenerated tissues.

The chondrogenesis of MSCs can be guided using growth factors [31] or biophysical/biomechanical stimuli [32] to improve the functional properties of the derived neo-cartilage tissues, including mature matrix formation [29,33]. Our previous study identified a unique population of chondrocyte precursors (CPs) derived from bone marrow mesenchymal stem cells (BMSCs) during chondrogenic induction [34]. These atelocollagen-embedded CPs (Kartigen^®^) can secrete glycosaminoglycan (GAG) and collagen type II but without lacunae formation.

A variety of biomaterials have been used for cartilage tissue engineering, including collagen [35], alginate [36], poly-lactic-glycolic acid [37], and tri-copolymer [38]. In this study, we selected atelocollagen because it is a low-immunogenic derivative of collagen obtained by the removal of N– and C–terminal telopeptide components [39]. Atelocollagen has been broadly applied in the regeneration of cartilage [34,40,41], intervertebral disc [42], cornea [43], periodontal tissues [44], and skin [45] to serve as a carrier for cell delivery and to provide an appropriate microenvironment for tissue regeneration.

A 9-year follow-up trial demonstrated that Kartigen^®^ integrated with the host tissue and resulted in the formation of hyaline-like cartilage, thereby improving the impaired knee functions [34]. Due to the lack of a randomized control group in the previous study, we initiated this controlled and randomized trial to evaluate the safety and efficacy of Kartigen for repairing cartilage defects in the weight-bearing site of medial femoral condyles through the comparison with the microfracture treatment.

## 2. Materials and Methods

### 2.1. Ethical Approval

This study was an open-label, controlled, randomized, single-center, phase Ⅰ clinical trial to evaluate the clinical safety of Kartigen^®^ and its clinical improvements versus microfracture. This study was approved by the Taiwan Food and Drug Administration (TFDA, study number 1076026300) and by the Institutional Review Board of Taiwan Adventist Hospital (IRB number: 105-B-09). According to the guidelines of the Declaration of Helsinki, informed consent was obtained from each subject.

### 2.2. Study Population

This study enrolled 15 patients with cartilage defects at the weight-bearing site of the medial femoral condyle. The size of cartilage defects ranged from 0.6 to 4 cm^2^. Patients were enrolled in this study between September 2018 and June 2020. Patients were selected according to inclusion and exclusion criteria in Table S1. They were randomly allocated into groups of Kartigen^®^ implantation (*n* = 10) or microfracture treatment (*n* = 5). Based on our previous study [34], the seeding density of CPs was 1.6–3.3 × 10^6^ cells/cm^2^, and the total cell number was less than 1.32 × 10^7^ cells. The schedule of study procedures and assessments is shown in Table S2.

### 2.3. Manufacture of Kartigen^®^

The manufacture of Kartigen*^®^* was described previously [34]. In brief, cell culture was performed under standard operative procedures at the cell processing unit (CPU) of Kartigen Biomedical Inc. (Taipei, Taiwan), following the Good Tissue Practice regulations. Fifteen mL of heparinized blood was aspirated from the iliac crest, collected in sterile 50-mL tubes. The MSCs were isolated using the density-gradient medium Ficoll (Cat. No. 17-5446-52, GE Healthcare, Little Chalfont, UK). The nucleated cells were collected from the interface, washed twice in PBS, and then suspended in Dulbecco’s Modified Eagle’s Medium (DMEM-LG, Cat. No. 31600, Gibco, Carlsbad, CA, USA) supplemented with the patient’s serum. BMSC were expanded to a sufficient number, enough to fill the cartilage defects of patients. The required cell number was estimated according to the size of the defects. Before the BMSCs were induced into CPs, a small portion of BMSCs were used to assess the number, viability, and the immunophenotype of BMSCs, and to undergo sterility testing. The cell number and viability were quantified using trypan blue staining and an automated cell counter. BMSC’s immunophenotype must be fulfilled by the minimal ISCT criteria: More than 95% of BMSCs are CD90-positive, and less than 2% of BMSCs are CD34-negative. Sterility testing must be negative for aerobic and anaerobic bacteria, mycoplasma, and <0.5 EU/mL endotoxin. The expanded cells were then seeded in excipient (Kartigen^®^) and cultured in CPs’ induction medium.

### 2.4. Surgical Operation

The knee lesions were assessed arthroscopically. The defect size was measured through the arthroscope. For the Kartigen^®^ implantation group, the required cell number of CPs was estimated before the collection of bone marrows. We aimed to implant 2 × 10^6^ cells/cm^2^ in the defect. The cartilage defect at the medial femoral condyle was approached either by mini-medial arthrotomy of 2 to 3 cm in length or by the arthroscope only. After debridement, the cartilage defect was filled with Kartigen^®^ and then sealed with fibrin glue (TISSEEL, Baxter AG). For microfracture, we followed a standard arthroscopic procedure as reported by Steadman et al. [46]. The multiple holes (microfractures) were about 3–4 mm apart, to preserve the structure and function of the subchondral plate.

### 2.5. Rehabilitation

Both groups of patients underwent the same rehabilitation regimen. The operated knee was kept in 20- to 30-degree flexion for 72 h, and then the knee was allowed to move freely. Partial weight-bearing was started 24 h after the operation, and full weight-bearing was allowed 4 weeks after the operation.

### 2.6. Safety Evaluation

A series of evaluations were assessed to assure the safety of the study for 2 years after treatments. These evaluations included physicians’ queries, physical examinations, laboratory tests, and radiological studies. The Common Terminology Criteria for Adverse Events was used to elicit and report toxic effects [47]. We specifically monitored the possible severe adverse events, including the severity of pain, infection of the operation site, joint adhesion, the abnormal gross appearance of the knee, active range of motion, loose body formation, and tumorigenesis.

### 2.7. Efficacy Evaluation

The preliminary efficacy was assessed using the International Knee Documentation Committee (IKDC) scoring system [48], visual analog scale (VAS), and arthroscopy. Arthroscopy was performed 1 year after the operation to observe the changes of defects. At the same time, a biopsy specimen (2 mm in diameter) was taken for histological analysis.

### 2.8. Arthroscopic and Histological Analysis

Second-look arthroscopy was done with the patients’ written consent. Alcian blue staining and immunohistology were applied to evaluate the existence of GAG and collagen type II. The International Cartilage Repair Society (ICRS) arthroscopic assessment scale was used to evaluate the degree of cartilage repair [49,50]. Alcian blue staining (Cat. No. B8438, Sigma-Aldrich, Saint Louis, MO, USA) was used to assess the amount of GAG accumulation in the implanted tissues. Nuclear Fast Red solution (Cat. No. H-3403, Vector Laboratories, Burlingame, CA, USA) was used for counterstaining. The immunohistochemistry was applied to quantify the protein expression level of collagen type II. An anti-collagen type II antibody (Cat. No. Ab34712, Abcam, Cambridge, UK) and UltraVision Quanto Detection System (Cat. No. TL-060-QHL, Thermo Scientific, Fremont, CA, USA) were used for the immunohistochemistry, according to the manufacturer’s instructions. All samples were processed and stained using the same procedure.

### 2.9. Statistical Methods

The statistical analyses of IKDC scores and VAS scores were performed using *t*-test and Wilcoxon signed-rank test, respectively. A two-tailed paired *t*-test was used to test the difference of IKDC scores at different time points (before and after the operation) of the same treatment group. To compare the efficacy of treatments, we used a one-tailed *t*-test to test the null hypothesis: IKDC scores of microfracture treatment were higher than that of Kartigen^®^ implantation. The statistical significance of the *t*-tests was set as *p* < 0.05. All statistical analyses were performed using Microsoft Excel.

## 3. Results

### 3.1. Demography

Fifteen patients were enrolled and randomized into two groups. The control group underwent microfracture, and the study group underwent Kartigen^®^ implantation. No patients were withdrawn from this trial. The flow chart of the study is shown in Figure S1. The demographic information of the patients is shown in Table 1. There was no significant difference in age between the two groups, but the defect sizes were significantly different.

### 3.2. Safety Assessment

In the Kartigen^®^ group, three patients exhibited three treatment-emergent adverse events (TEAEs): renal stone with hematuria, cervical spondylosis, and upper respiratory tract infection, respectively. In the control group, two patients demonstrated four TEAEs, including urinary tract infection, cataract, prostate hypertrophy, and coronary artery stenosis. All seven TEAEs were not treatment-related. These TEAEs are summarized in Table S3. By X-ray examination and MRI study, there was no inflammation, joint adhesion, loose body, or tumorigenesis in the Kartigen^®^-implanted knee and microfracture-treated knee. Physical examination and laboratory tests revealed no infection among the 15 patients. Moreover, neither mortality nor complications were noted after operations in this study. Some laboratory values were abnormal, such as blood sugar, GOT, GPT, and urinary red blood cell in both groups. However, they were felt to be reflective of each patient’s underlying medical condition and not as results of participation in the study.

### 3.3. Efficacy Assessment

**Figure 1:**
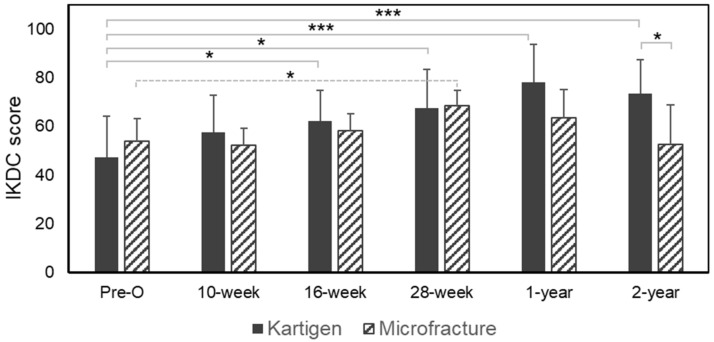
Assessment of knee functions of daily activities by IKDC scores. The knee functions of the patients were significantly improved 16 weeks after the Kartigen^®^ implantation and 28 weeks after microfracture, respectively. The paired *t*-test was conducted to determine the difference between the means of the IKDC scores before and after the treatments at different time points among the same groups. Two years after operation, the IKDC score of the Kartigen^®^ group was higher than the control group. The error bars stand for standard deviations. Asterisks represent the statistical significance: * *p* < 0.05, *** *p* < 0.005.

We applied IKDC subjective knee evaluation criteria to evaluate the knee function before and after the treatments. Figure 1 showed a tendency that the IKDC scores continuously improved in the Kartigen^®^ group, but the control group achieved the highest IKDC score in the 28th week and gradually decreased. In the study group, the IKDC score before Kartigen^®^ implantation was 47.1 ± 17.0 (mean ± standard deviation) and then significantly improved to 62.1 ± 12.8 (*p*-value = 0.025) 16 weeks after implantation. The IKDC scores continuously increased to 78.2 ± 15.4 with a statistical significance (*p*-value < 0.005) one year after the operation and still maintained at 73.6 ± 13.8 2 years after the operation. While IKDC scores slightly decreased in the second year, the scores were still significantly higher than that of preoperation (*p*-value < 0.005). Meanwhile, although the control group demonstrated significantly improved IKDC scores from 54.0 ± 9.1 (preoperation) to 68.5 ± 6.1 (28 weeks after the operation) with a *p*-value of 0.032, the score decreased to 63.5 ± 11.6 1 year after the operation and to 52.6 ± 16.4 2 years after the operation. The comparison of IKDC scores between the Kartigen^®^ group and the microfracture group in the second year exhibited statistical significance with the *p*-value of 0.029. Still, a longer follow-up will be needed to evaluate the long-term efficacy and longevity of the regenerated cartilage.

**Figure 2:**
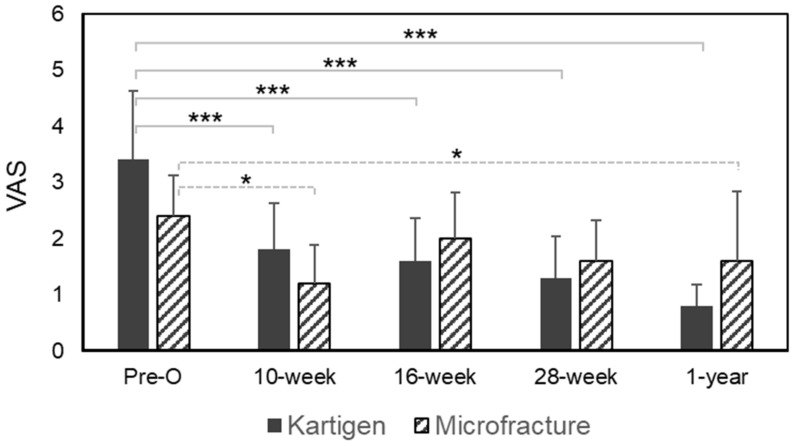
VAS assessment. The pain relief was effective and significant in the Kartigen^®^-treated group, as VAS scores decreased since the 10th week after operation, but the VAS scores flatulated in the control group. The error bars stand for standard deviations. Asterisks represent the statistical significance: * *p* < 0.05, *** *p* < 0.005.

The VAS was used to measure the pain before and after treatments. Both the Kartigen^®^ implantation and microfracture treatment effectively achieved pain relief at the 10th week after the operation (Figure 2). However, the Kartigen^®^ implantation exhibited consistent pain relief through the 1-year follow-up, which was validated statistically using Wilcoxon signed-rank test with *p*-values < 0.005.

### 3.4. Arthroscopy and Histological Analysis

**Figure 3:**
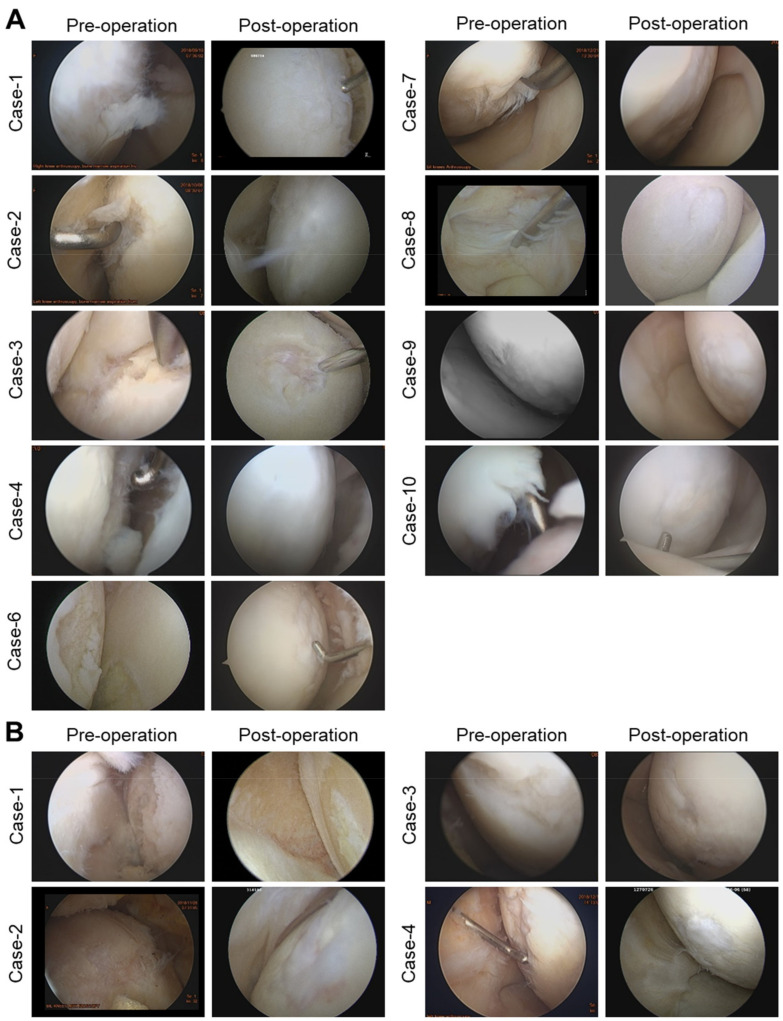
Arthroscopic images before and 1 year after operation. Images of Kartigen^®^ group (**A**) and microfracture group (**B**).

The regeneration of cartilage defect was grossly observed by knee arthroscopy, and a specimen of 2 mm in diameter was taken 1 year after surgery. Nine out of 10 patients in the Kartigen^®^ group and 4 out of 5 patients in the control group consented to second-look arthroscopy. The gross appearance of regenerated cartilage tissues was examined under arthroscope and compared with their corresponding arthroscopic images taken before treatment (Figure 3). Eight out of 9 of the Kartigen^®^ group exhibited smooth and elastic surfaces at the recipient sites (Figure 3A), but none of the control group showed normal or near-normal appearance. Only fibrillated surface was noted (Figure 3B). The ICRS scale is shown in Table 2 with the classification of grade I (normal tissue), grade II (nearly normal tissue), grade III (abnormal), grade IV (severely abnormal).

**Figure 4:**
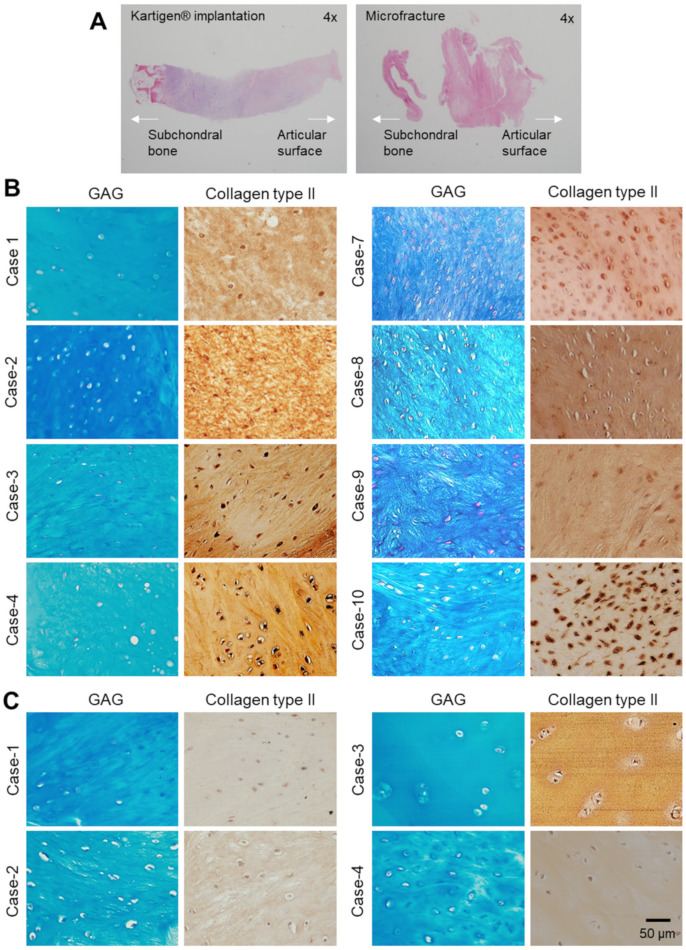
Immunohistochemical analyses of biopsy specimens. (**A**) Whole-slide histological images of regenerated cartilage under 4× objective lens. Alcian blue staining and immunochemical study show expression levels of GAG/Collagen type II, respectively, in Kartigen group (**B**) and microfracture group (**C**).

The Kartigen^®^ group demonstrated better results in both arthroscopic observations and histological analysis (Figure 4). In the control group, the regenerated cartilage did not exhibit columnar chondrocyte distribution but only fibrocartilage (Figure 4A). GAG and collagen type II were observed in the biopsy specimens, and a total of 12 specimens (Kartigen^®^, *n* = 8; control, *n* = 4) are presented in Figure 4B,C. New chondrocytes at defect had the same appearance as the original ones (Figure 4B). However, the chondrocytes in the repaired defect in the Kartigen^®^ group were smaller and denser than those in the original cartilage.

## 4. Discussion

The development of Kartigen^®^ was started from our previous study using atelocollagen-embedded chondrogenic BMSCs to repair the full thickness of cartilage defect in miniature pigs [51]. The promising results encouraged us to initiate a case series study to test the safety and efficacy of Kartigen^®^ for treating cartilage defects. In the 9 years of follow-up study, improvement of knee functions was satisfactory and sustainable [34]. Most importantly, infection, inflammation, joint adhesion, loose body, and tumor formation were not found in the Kartigen^®^-implanted knees. However, in the case series study, the contralateral knees of the same patients were used as a control group. To further validate the therapeutic efficacy of Kartigen^®^ in comparison with standard surgical treatments, such as microfracture, a new controlled and randomized phase I clinical trial was conducted. As expected, no severe adverse events were found, and all the TEAEs were not treatment-related. Although clinical outcome was similar for Kartigen implantation and microfracture in 1-year follow-up, a sustained improvement of knee functions was found in the Kartigen^®^ group in the 2 years follow-up.

Microfracture was introduced for focal articular cartilage repair in the 1980s and soon became a standard treatment for cartilage defects [9,46,52]. However, its results are usually associated with fibrocartilage production [12,13], and its long-term efficacy remains poor [16,53]. Mosaicplasty provides a better improvement than microfracture, but there are still several disadvantages, such as the donor-site morbidity for autografts [54], limited sources of grafts, a technical challenge in leveling the graft during operation [55], and poor integration of the implant into host tissue [56,57]. On the contrary, the procedure of Kartigen^®^ implantation is technically simple and can be carried out with a small arthrotomy or using arthroscope. Furthermore, there is no donor site morbidity, and sources of cells are not limited.

The ability of engrafted cells to integrate into the recipient site and participate in the repair process is crucial for successful clinical outcomes. It has been reported that intra-articular injection of autologous MSCs reduced the degeneration of cartilage defects and provided pain relief [58], but integration of injected MSCs into damaged cartilage defects is unclear and doubtful. On the contrary, using atelocollagen as a cell carrier, Kartigen^®^ prevents cell loss, is easy for implantation for cartilage defects of any shape, and achieves uniform cell distribution at the recipient site. These advantages contribute to the efficacy and durability of Kartigen^®^ implantation. In addition, atelocollagen has been proven to enable a gradual proliferation and matrix synthesis of chondrocytes, which allow chondrocytes to maintain their phenotype for up to 4 weeks in vitro [40]. Atelocollagen gel can also support cell proliferation, matrix synthesis, and chondrogenic differentiation of MSCs [42]. Recently, the Adachi group reported the efficacy of repairing osteochondral defects with minced cartilage embedded in atelocollagen gel [41]. Our previous clinical trial with 9 years of follow-up also demonstrated the safety and efficacy of atelocollagen in repairing cartilage defects together with CPs [34].

Unlike previous studies using mature chondrocytes for cartilage repair in ACI [59], our study showed CPs in Kartigen^®^ exhibiting sufficient integration capacity. Under arthroscopic examination, the integration between the graft and the recipient site was complete (Figure 3). The histological analyses of the biopsy specimens also demonstrated the integration of the implanted tissue into the surrounding articular cartilage (Figure 4). The accumulation of GAG and collagen type II was confirmed in the biopsy specimens (Figure 4). However, there are several limitations to this study. Because this is a phase I study, the sample size number is small. The 2 years of follow-up are not long enough to reach a final conclusion for cartilage defect repair.

Recent advances in 3D bioprinting [60,61,62,63] have enabled reconstructions of functional living cartilage to recapitulate the complexity and architecture [64,65] of an articular surface. We are looking into partnerships to integrate that technology and potentially further improve the outcome with Kartigen^®^ implantation. This may result in more favorable biomechanical properties at the recipient sites and allow earlier weight-bearing and range of motion without concern of graft detachment. Since MSCs are multipotent, additional biochemical and biomechanical stimulations can delicately manipulate the chondrogenic differentiation and maturation of seeded cells [66,67] to improve the functional properties of the derived neo-cartilage tissues [29]. The integration of 3D bioprinting and biomimetic in vitro chondrogenesis will drive advanced therapeutic innovations for cartilage repairs.

## 5. Conclusions

In this study, Kartigen^®^ containing CPs was proven safe and free of adverse events, such as infection, inflammation, joint adhesion, loose body, or tumor formation.

## Figures and Tables

**Table 1:** Demography of the patients.

Group	Kartigen^®^ Group (*n* = 10)	Microfracture (*n* = 5)
Gender (F:M)	5:5	2:3
Age (year)	22–77 (54.8 ± 18.0)	55–77 (67.8 ± 8.5)
Defect size (cm^2^)	1.3–4.0 (2.9 ± 0.8) *	0.6–1.5 (1.0 ± 0.4)
Defect treatment	Kartigen^®^	Microfracture

* Note: The defect size of the Kartigen^®^ group was significantly larger than that of microfracture group (*p* < 0.005).

**Table 2:** ICRS arthroscopic assessment.

ICRS Cartilage Repair Assessment			
Kartigen^®^ Group (*n* = 10)		Microfracture Group (*n* = 5)	
Grade I	3	Grade I	0
Grade II	5	Grade II	0
Grade III	1	Grade III	3
Grade IV	0	Grade IV	1
Total	9	Total	4

## Supplementary Materials

The following are available online at https://www.mdpi.com/article/10.3390/polym13183029/s1. Table S1: Inclusion and exclusion criteria, Table S2: Scheduled visits of the study design, Table S3: Adverse events, Figure S1: Flowchart of this clinical trial.
