# Supplementary material for: Safety and Efficacy of Kartigen® in Treating Cartilage Defects: A Randomized, Controlled, Phase I Trial
Source: Polymers (Basel). 2021 Sep 7;13(18):3029. doi: 10.3390/polym13183029 (PMC8466236; doi:10.3390/polym13183029)
Supplement: Supplementary file 1 [file polymers-13-03029-s001.zip › polymers-1341150-supplementary.pdf]

polymers

MDPI

Article

Safety and Efficacy of Kartigen® in Treating Cartilage Defects: A Randomized, Controlled, Phase I Trial

Yen-Liang Liu [1,2], Chun-Che Yen [3], Tzu-Shang Thomas Liu [4], Chih-Hung Chang [5,6], Tiffany Ting-Fang Shih [7], Jyh-Horng Wang [8], Ming-Chia Yang [9], Feng-Huei Lin [10] and Hwa-Chang Liu [8,11,*]

1 Master Program for Biomedical Engineering, College of Biomedical Engineering, China Medical University, Taichung City 406040, Taiwan; allen.liu@cmu.edu.tw
2 Graduate Institute of Biomedical Sciences, College of Medicine, China Medical University, Taichung City 406040, Taiwan;
3 Kartigen Biomedical Inc., Taipei City 100047, Taiwan; cyenslk@gmail.com
4 Southern California Bone and Joint Clinic, Apple Valley, California 92307, USA; redcometsports@gmail.com
5 Department of Orthopaedic Surgery, Far Eastern Memorial Hospital, New Taipei City 220216, Taiwan; orthocch@mail.femh.org.tw
6 Graduate School of Biotechnology and Bioengineering, Yuan Ze University, Taoyuan City 320315, Taiwan
7 Department of Medical Imaging and Radiology, National Taiwan University Hospital, Taipei City 100225, Taiwan; ttfshih@ntu.edu.tw
8 Department of Orthopaedic Surgery, National Taiwan University Hospital, Taipei City 100225, Taiwan; jhwang@ntuh.gov.tw (J.H.W.), hcliu@ntuh.gov.tw (H.C.L.),
9 Biomedical Technology and Device Research Laboratories, Industrial Technology Research Institute, Hsinchu 310401, Taiwan; s1979329@gmail.com
10 Department of Biomedical Engineering, College of Engineering, National Taiwan University, Taipei City 106319, Taiwan; double@ntu.edu.tw
11 Department of Orthopaedic Surgery, Taiwan Adventist Hospital, Taipei City 105404, Taiwan
* Correspondence: hcliu@ntuh.gov.tw

Citation: Liu, Y.-L.; Yen, C.-C.; Liu, T.-S.T.; Chang, C.-H.; Shih, T.T.-F.; Wang, J.-H.; Yang, M.-C.; Lin, F.-H.; Liu, H.-C. Safety and Efficacy of Kartigen® in Treating Cartilage Defects: A Randomized, Controlled, Phase I Trial. *Polymers* **2021**, *13*, 3029. https://doi.org/10.3390/polym13183029

Academic Editors: José Miguel Ferri, Vicent Fombuena Borràs and Miguel Fernando Aldás Carrasco

Received: 30 July 2021
Accepted: 31 August 2021
Published: 7 September 2021

Publisher's Note: MDPI stays neutral with regard to jurisdictional claims in published maps and institutional affiliations.

Copyright: © 2021 by the authors. Licensee MDPI, Basel, Switzerland. This article is an open access article distributed under the terms and conditions of the Creative Commons Attribution (CC BY) license (http://creativecommons.org/licenses/by/4.0/).

Table S1 | Inclusion and Exclusion Criteria

Inclusion criteria	Exclusion criteria
1. Subjects are conscious, able to express, and capable to communicate with Mandarin and Taiwanese<br>2. Subjects who have been diagnosed with medial femoral condyle cartilage defect (diagnosed by knee X-ray examination and knee MRI)<br>3. Age between 20 and 80 years<br>4. Subjects with knee varus deformity less than 10 degree (i.e. femorotibial angle less than 190 degree)<br>5. Willing to participate this trial and capable to provide signed informed consent before study<br>6. Single defect between 0.36 and 4 cm2 confirmed by knee arthroscopy (if both knees are defective, the larger or the more painful knee was chosen to participate this trial).	Subjects with immature bone<br>Subjects with severe degenerative arthritis, and artificial joint replacement is deemed necessary<br>Subjects with other injuries, such as knee meniscus injury, or cruciate ligament defect<br>Subjects with arthritis (such as rheumatoid arthritis, gout arthritis), or knee articular infection, such as gout, pseudogout, primary Paget disease, or any chronic pain syndrome<br>Subjects who are sensitive to the active ingredient or excipient of fibrin glue product (Baxter Tisseel Solution for sealant)<br>Subjects who are long-term bed-ridden for more than three months<br>Subjects who continuously use steroid 5mg/day for more than two weeks<br>Alcoholism<br>Subjects who are not compliant to clinical protocol<br>Subjects with infectious diseases, such as acquired immune deficiency syndrome (AIDS), hepatitis B virus (HBV), hepatitis C virus (HCV), or carrier of syphilis.<br>Subjects with any cognitive or psychiatric illness<br>Subjects with severe knee sigmoid ligament bursitis, acute articular injury, or completed knee articular cartilage defect<br>Subjects who are injected with steroid via intravenous, intramuscular or articular<br>Subjects who are suffered with autoimmune disease and received immunosuppressive treatments (such as alefacept, etanercept, infliximab or cyclosporine)<br>Female subjects who are lactating or pregnant.<br>Female subjects with reproduction ability are not able to use contraception methods (Subject is suggested to use contraception method, such as using intrauterine device during the study)<br>Subjects who had received knee articular surgery (except the knee arthroscopy examining surgery)<br>Subjects who had received cell therapy<br>Subjects who have participated in other investigational study within 1 month prior screening<br>Subjects not suitable to participate this as judged by the investigator<br>MRI contraindications: subject with heart pacemaker, artificial heart valve, carotid surgery with vascular clipping or stent, cochlear implant, IVC filter, Swan-Ganz catheter, metallic foreign body around his/her eye(s), implanted insulin or other infusion pump, implant defibrillator, nerve stimulator, surgical metallic implants or claustrophobia.<br>Subjects with body mass index (BMI) > 35<br>Subjects with malignancy<br>Multiple chondral defects; or the single chondral defect small than 0.36 cm^2, or larger than 4 cm^2 examined by knee arthroscopy

Table S2 |

	Screen-ing		Dosing			Evaluation and follow-up						
Visit	1	2	3	3-1	3-2	4[6]	5	6	7	8	9	10
Week	0	0	1	5	6	7[6]	10	12	16	20	28	56
ICF	X											
Demographic data	X											
Medical history	X											
Knee X-ray[1]	X						X		X		X	X
Knee MRI[2]	X										X	X
IKDC questionnaire	X						X		X		X	X
Chest X-ray	X											
Electrocardiogram	X											
Laboratory tests[3]	X						X		X		X	X
Knee arthroscopy		X										X
Microfracture[4]		X										
Randomization		X										
Bone marrow drawn[5]		X										
Stitches removal			X									
Kartigen® implantation[5]				X								
Physician's query			X		X	X	X	X	X	X	X	X
Physical examination	X						X		X		X	X
ICRS scores		X										X
Record AEs	X		X	X	X	X	X	X	X	X	X	X
Concomitant medica-tions	X	X	X	X	X	X	X	X	X	X	X	X
Exit study												X

Scheduled visits of the study design

1. Knee X-ray was captured from front, side and merchants view.
2. Contrast agent is not required for knee MRI.
3. Laboratory tests include biochemistry, hematology tests (complete blood count, serum blood and blood biochemistry) and urine tests.
4. Only subjects in control group were performed arthroscopic microfracture.
5. Only subjects in experimental group were drawn for bone marrow and injected with Kartigen®.
6. Visit 4 was in week 3 in control group, rather than week 7.

Table S3 | Adverse Events

Subject ID /Treatment	Age (y) /Sex	AE term	Body System	TEAE	Severity	Relation-ship	Outcome	Action Taken	SAE
Case 1 Kartigen	69.4 Female	Other (Hematuria, Right renal stone)	Urinary system	Yes	Grade 1	Not Related	Recovered/Resolved	CT of abdomen, medical treatment, urine cytology, Renal sonography, cystoscopy. OPD follow-up.	No
Case 5 Kartigen	68.9 Female	Other (Cervical spondylosis C4/C5, decreased disc space C6/C7)	Nervous system	Yes	Grade 1	Not Related	Recovering/Resolving	X-ray, MRI	No
Case 7 Microfracture	76.9 Female	Other (Urinary Tract Infection)	Urinary system	Yes	Grade 1	Not Related	Recovered/Resolved	Urine culture, Antibiotic medical treatment, OPD follow-up	No
		Other (Cataract)	Nervous system	Yes	Grade 1	Not Related	Recovered/Resolved	Slit-lamp microscope, Topical eye drops	No
Case 9 Microfracture	67.5 Male	Other (Prostate hypertrophy)	Reproductive system	Yes	Grade 1	Not Related	Recovering/Resolving	medical treatment, OPD follow-up	No
		Other (Coronary artery stenosis)	Cardiovascular system	Yes	Grade 1	Not Related	Recovering/Resolving	Anticoagulant (TAH)、Cardiac catheterization (Transfer to National Taiwan University Hospital for treatment)	No
Case 14 Kartigen	59.1 Female	Other (Upper respiratory tract infection)	Respiratory system	Yes	Grade 1	Not Related	Recovered/Resolved	medical treatment	No

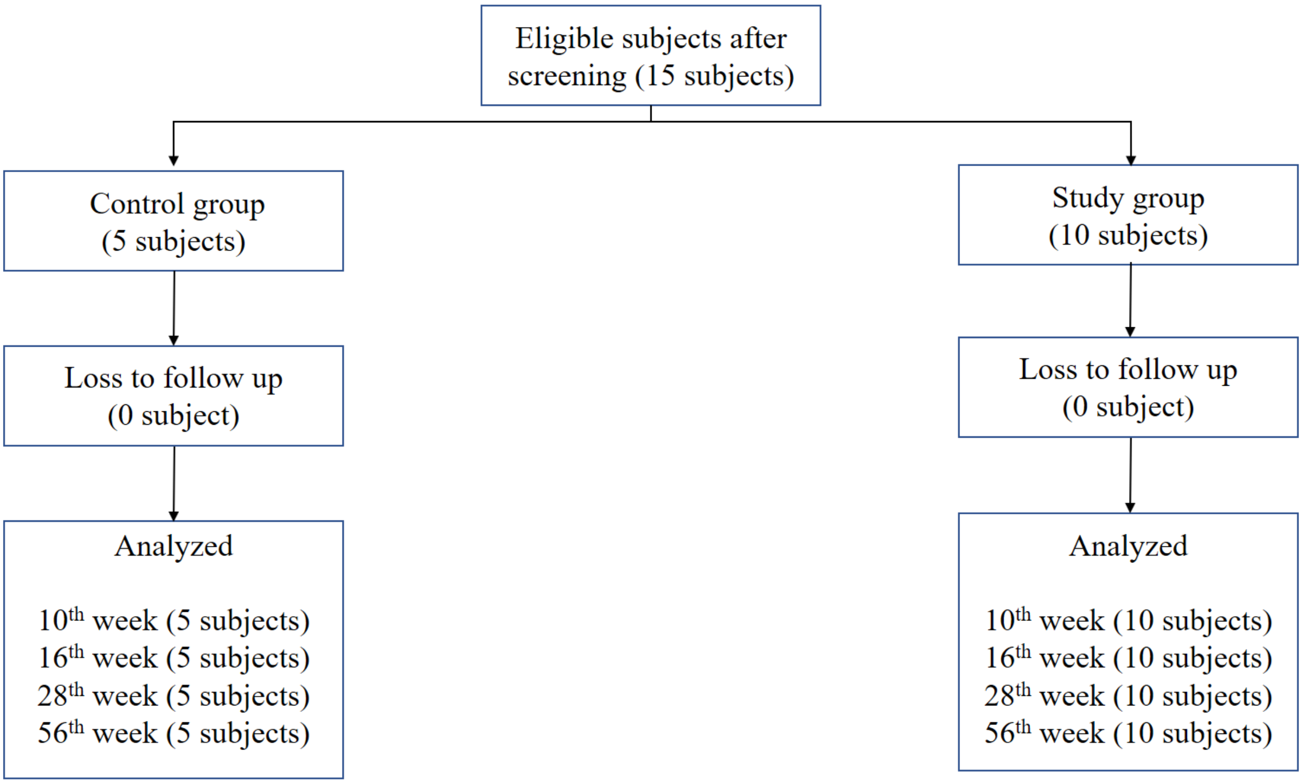

Figure S1 | Flowchart of this clinical trial.
